# A visual illusion that influences perception and action through the dorsal pathway

**DOI:** 10.1038/s42003-019-0293-x

**Published:** 2019-01-28

**Authors:** Cristina de la Malla, Eli Brenner, Edward H. F. de Haan, Jeroen B. J. Smeets

**Affiliations:** 1 Vision and Control of Action (VISCA) Group, Department of Cognition, Development and Psychology of Education, Institut de Neurociències, Universitat de Barcelona, Passeig de la Vall d’Hebron 171, 08035 Barcelona, Catalonia Spain; 2 Department of Human Movement Sciences, Institute for Brain and Behavior Amsterdam, Vrije Universiteit Amsterdam, Van der Boechorststraat 9, 1081BT Amsterdam, The Netherlands; 3 Department of Psychology, Amsterdam Brain and Cognition, University of Amsterdam, Nieuwe Achtergracht 129, 1001NK Amsterdam, The Netherlands

## Abstract

There are two main anatomically and physiologically defined visual pathways connecting the primary visual cortex with higher visual areas: the ventral and the dorsal pathway. The influential two-visual-systems hypothesis postulates that visual attributes are analyzed differently for different functions: in the dorsal pathway visual information is analyzed to guide actions, whereas in the ventral pathway visual information is analyzed for perceptual judgments. We here show that a person who cannot identify objects due to an extensive bilateral ventral brain lesion is able to judge the velocity at which an object moves. Moreover, both his velocity judgements and his interceptive actions are as susceptible to a motion illusion as those of people without brain lesions. These findings speak in favor of the idea that dorsal structures process information about attributes such as velocity, irrespective of whether such information is used for perceptual judgments or to guide actions.

## Abstract (web summary)

Cristina de la Malla et al. show that an individual with extensive lesions to the ventral visual pathway can accurately judge the velocity at which an object moves despite not being able to correctly identify the object in question. This findings support a role of the dorsal visual pathway in judging velocity irrespective of perceptual processing.

## Introduction

Two main visual pathways have been identified in the human brain: one going from the primary visual cortex to the inferior temporal cortex via V4 (ventral pathway) and one from the primary visual cortex to the posterior parietal cortex via the middle temporal visual area (MT, dorsal pathway)^1^. The separation was originally ascribed to a distinction between the processing of attributes related to object identity and object location, respectively^2^. We will refer to this distinction as the attributes hypothesis. The very influential two-visual-systems hypothesis^3^ later elaborated on this by linking the ventral and the dorsal pathways to independent processing of information for different purposes: for action and perception.

According to the two-visual-systems hypothesis, all visual attributes are processed twice: in the ventral pathway for recognizing objects (perception) and in the dorsal pathway for guiding one’s hand towards them (action)^4^. This claim is based on the notion that in order to perform an action such as grasping a cup, people primarily need information about the cup’s precise position with respect to themselves. This information changes whenever they move. In contrast, in order to identify the cup as being their own cup, they need a detailed analysis of its characteristics such as its color, size, and shape. They will mainly rely on information that is independent of their position with respect to the cup, but might take information from the environment into account such as that the cup is lying next to the book they were reading. Such differences between guiding actions and recognizing objects calls for different analyses of the available information, which according to the two-visual-systems hypothesis occurs separately for perception and action tasks in the two-visual pathways. According to the original attributes hypothesis, each attribute is processed in only one of the pathways, irrespective of whether it is being used for perception or action.

Despite the fundamental difference between the attributes and the two-visual-systems hypothesis in terms of whether or not the same attribute is processed twice, distinguishing between the two hypotheses is difficult, precisely because recognizing objects often requires information about different attributes than does manipulating them. The initial evidence favouring the two-visual-systems hypothesis was based on two types of clinical cases: individuals with visual form agnosia such as DF, who could not report the dimensions or orientations of objects but could successfully interact with them^5,6^, and individuals with optic ataxia, who could judge objects’ shapes but could not successfully interact with them^6,7^. This evidence has been questioned on the grounds of psychophysical^8–10^, neuropsychological^11,12^, and neurophysiological^13,14^ data. More importantly, clinical evidence does not show that information about the same attribute is processed separately for perception and for action, it only shows that sometimes certain attributes cannot be used for one or the other^15^.

The second line of evidence for visual information being processed differently for perception than for action is based on studies on the effects of visual illusions on actions. According to the two-visual-systems hypothesis, perceptual illusions originate in the ventral pathway, where all information that could help make perceptual judgments is taken into account. Thus, illusions should not affect actions. Many studies have compared the influence of size illusions on the maximal grip opening when reaching to grasp objects with their influence on perceptual judgments of the objects’ sizes^8,16,17^. Other actions that have been compared with perceptual judgments of size or distance include saccades^18–20^, manual tracking^21^, lifting^17,22^, pointing^23,24^, bimanual grasping^25,26^, and stepping^27,28^. Some authors interpret the results as support for the two-visual-systems hypothesis, whereas others do not^10,29–31^. One reason why the same findings can be interpreted in different ways is that illusions often only influence a specific attribute, and there is no consensus about the attributes that are used to guide some actions^15^.

Until now, studies have mainly examined illusions of attributes such as size that, according to what we call the attributes hypothesis, are processed in the ventral pathway. A prototypical example of an attribute that is processed in the dorsal pathway is motion^32^. One way to create motion illusions is by embedding motion within a target. Embedded motion has been shown to affect a static target’s apparent position^33–36^, and a moving target’s apparent motion direction^37,38^ and speed^39,40^. We previously reported that in contrast to what the two-visual-systems hypothesis would predict, embedded motion led to errors in interception that were equivalent to the illusory changes in the perceived target motion^40^. This finding supports the idea that the dorsal pathway is essential for all aspects of motion processing, regardless of whether one only needs to perceive the object or needs to interact with it.

Finding that an individual with damage to the ventral pathway (such as DF) can judge a target’s velocity would provide evidence for the idea that the dorsal pathway is involved in all aspects of motion processing. Finding that such an individual’s perceptual judgments and actions are both susceptible to a motion illusion would confirm that the same pathway is responsible for both kinds of tasks. To explore this, such an individual with a ventral lesion (MS) and an age-matched control (BK) participated in a slightly modified version of an experiment that we recently conducted with ten young participants^40^. We show that MS’s goal-directed movements are affected by a visual illusion that changes the target’s apparent velocity. Furthermore, his judgments about how fast the target moves are as susceptible to the illusion as are those of control participants. This shows that the effect of the illusion on both tasks must originate in the dorsal pathway. Our results therefore provide evidence against separate processing of information for perception and action within two segregated pathways^3^. They speak in favor of the dorsal pathway being specialised for processing attributes that are likely to be used for the on-line control of movement^2^.

## Results

### Participants and task

MS is a 65-year-old male who suffered bilateral damage to temporo-occipital regions in 1970^41^. As a result, he has a severe agnosia for objects^42^ but can perceive motion^43^ and has corrected to normal Snellen acuity in both eyes. The age-matched control (BK) is a 63-year-old female with normal vision and without known history of brain damage. Due to his object agnosia and co-morbid impairments, explaining the tasks to MS was more difficult than usual. Moreover, he often needed to be reminded to keep his eyes on the fixation point.

**Fig. 1:**
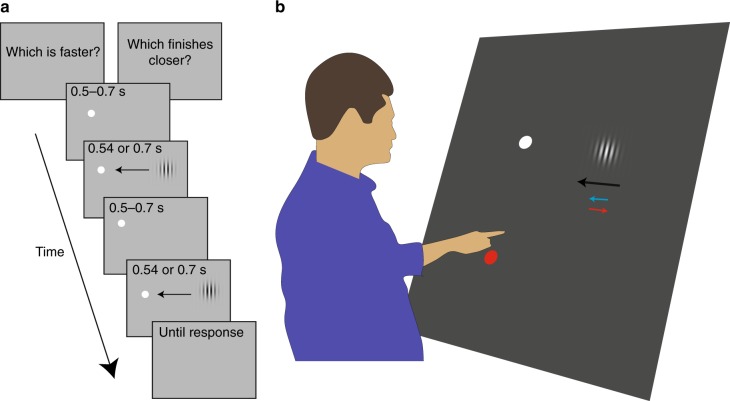
Schematic representation of the tasks. Participants were asked to fixate the white dot. In separate blocks they either **a** judged which of two sequentially presented patches moved faster or disappeared closer to the fixation point or **b** tried to tap on the patch as it passed the fixation point. Targets moved horizontally (black arrow) towards the fixation point. They could have embedded motion in the same direction as the patch (blue arrow) or in the opposite direction than the patch (red arrow)

Our experiment consisted of three tasks (performed in separate blocks): two in which we evaluated the ability to judge the target’s velocity and position relative to the fixation point 100 ms before the target reached this point (Fig. 1a, see Methods for further details), and one in which we evaluated the ability to tap on the target when it reached the fixation point (Fig. 1b). The target was a moving Gabor composed of a vertical sine wave grating of which the contrast was modulated by a two-dimensional Gaussian. The Gabor patches moved horizontally towards the fixation point at a constant velocity of either 40 or 50 cm/s (black arrow in Fig. 1b). The grating also moved horizontally within the Gaussian that defined the patches. It moved at 10 cm/s, either in the same direction as the Gaussian (blue arrow in Fig. 1b) or in the opposite direction (red arrow in Fig. 1b).

### Perceptual judgments about moving targets

**Fig. 2:**
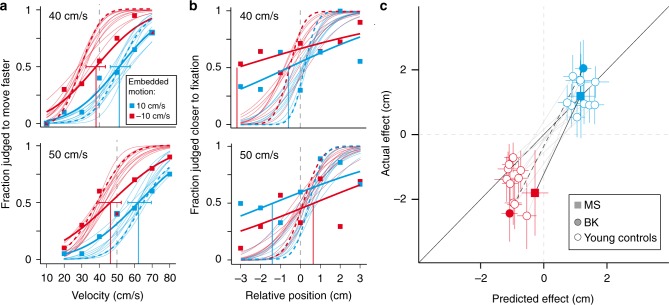
Influence of embedded motion. Effect of the embedded motion on **a**   the judged velocity, and **b** the judged position. **a**, **b** Squares: fraction of trials on which MS judged the comparison patch to move faster or to disappear closer to the fixation point than the standard patch. Thick curves: fitted psychometric functions. The psychometric curves for controls are plotted for comparison (dashed curves: BK; thin curves: young controls, adapted from ref. ^40^). **c** The effect of embedded motion on tapping errors as a function of the effect predicted from the velocity judgments (positive is in the direction of target motion). A set of two symbols connected by a grey line represents an individual subject’s values for the two directions of embedded motion, averaged across the two velocities of the standard patch (see Methods). Filled squares: data for MS; disks: data for controls (filled: BK, open: young controls, adapted from ref. ^40^). All error bars are 95% confidence intervals for each point of subjective equality (PSE) calculated using parametric bootstrapping with 1000 samples

Although MS was unable to report any characteristics of the target, judging in which of two intervals the target moved faster was remarkably effortless. The direction of the embedded motion had a clear effect on MS’s perceived velocity (separation between blue and red curves in Fig. 2a). When embedded motion was in the same direction as that of the target, the target was perceived to move faster than it really was (blue curve). When embedded motion was in the opposite direction than that of the patch, the target was perceived to move slower than it really was (red curve). The mean influence of the 20 cm/s difference in embedded motion was 14.6 cm/s. MS’s reports in judging the velocity were twice as variable as BK’s, as is visible in the difference in steepness of the thick and dashed curves in Fig. 2a (standard deviation in velocity of 24 and 12 cm/s, respectively). However, MS’s reports were not much more variable than those of some of the young controls (range 11–20 cm/s).

The position task was almost impossible for MS to complete. He reported finding it very difficult to identify which of two subsequent intervals contained the target that disappeared closer to the fixation point, so testing was stopped after 314 of the 560 planned trials. His difficulties are reflected in the very shallow slopes of his psychometric functions in Fig. 2b. BK could complete such a task without problems, as the dashed psychometric curves show. MS’s average standard deviation was 5.9 cm, whereas BK’s was 0.6 cm, which is even better than the best young control (range 1.0–1.6).

### Interception of moving targets

Despite his difficulties in reporting patches’ relative positions, MS could perform the interception task. Embedded motion influenced his tapping errors (vertical position of the squares in Fig. 2c) in much the same manner as it did for the controls. We compared the errors that participants made in trying to hit the targets with what one would expect based on an extrapolation of 100 ms using the patches’ judged velocities (see Methods). The errors in perception led to equivalent errors in interception (symbols close to unity line). MS’s tapping performance was mainly more variable than that of BK and the young controls (standard deviation of 4.5 cm, compared with 2 cm for BK and a range from 1.1–2.5 cm for the young controls).

## Discussion

Two aspects of our results are in clear conflict with predictions derived from the two-visual-systems hypothesis. Firstly, the fact that MS could report object motion so well despite a severely damaged ventral pathway shows that an intact ventral pathway is not needed to process motion information for perception. Secondly, we found that our motion illusion affected perception and action to the same extent in MS as in controls. Since the dorsal pathway includes MT^2^, which is known to process visual information about motion, our results support the idea that the dorsal pathway processes the information about motion for both perception and action. One might argue that the illusion originates at an early stage of visual processing, before the two pathways separate^44^. However, even if the illusion were to originate before the separation, its influence on perception cannot be mediated by the ventral pathway in MS because his ventral pathway is severely damaged. Finding an influence on both perception and action is also consistent with the illusion being the result of an interaction between local and global motion signals in areas further down the dorsal pathway than MT^45^.

We are not the first to suggest that the dorsal stream is involved in perception tasks. Interactions between the ventral and the dorsal pathways have been acknowledged by many authors^9,12,46–50^, including Goodale and Milner^4^, who stated that the ultimate role of the ventral stream is also to serve action rather than perception per se. Rizzolatti and Matelli^51^ established a subdivision within the dorsal stream: a dorso-dorsal stream, which includes areas V6, V6A and medial intraparietal areas (MIP), and a ventro-dorsal stream, including MT and visual areas of the inferior parietal lobule. According to their description, the ventro-dorsal stream is involved both in perception and in action, while the dorso-dorsal stream is specifically involved in motor tasks. The role of MT in the ventro-dorsal stream that follows from this subdivision is in line with our results on motion perception and tapping errors.

MS’s tapping performance is consistent with his perceptual performance. MS was about twice as variable as controls in both his speed judgments and his tapping performance. Thus, MS’s perceptual speed judgments are not more strongly affected by the damage to his ventral pathway than are his actions, contrary to what one would expect according to the two-visual-systems hypothesis. Moreover, his tapping performance is also much more variable than that of controls, which is inconsistent with the ventral pathway not being involved in action. The relatively steep slopes of the lines in Fig. 2c for both MS and BK may be the result of us having underestimated the predicted effect. The predicted magnitude of the illusion’s effect on interception depends on the time for which the motion has to be extrapolated (the visuomotor delay). We are likely to have underestimated the visuomotor delay for MS and BK because we did not consider the fact that the visuomotor delay is known to increase slightly with age^52^. However, this cannot explain the whole pattern, as for MS the predicted error for embedded motion in the opposite direction than the patch’s motion is mainly small because of his small velocity judgment errors in this condition.

Judging the positions relative to the fixation point was particularly difficult for MS. This was not the case for BK (who even outperformed young controls), which allows us to rule out the possibility of age being responsible for MS’s difficulties in this task. Although MS’s judgments are not ‘random’, they are ten times less precise than those of the controls. He may have been unable to judge the position of the target relative to the fixation point because relative positions might be processed in the ventral pathway. He might thus have been forced to rely exclusively on judgments of the endpoint positions relative to himself, which is presumably processed in the dorsal pathway. Although the illusion that we used is known to influence judgments of both velocity and position, the influence on the perceived velocity is much larger, so we could compare the interception data with the velocity judgments, ignoring any influence on the perceived position. This was also true for MS, whose judgments were more variable but who did not seem to be influenced to a different extent by the illusion in any of the tasks.

Lisi and Cavanagh^37,38^ reported that embedded motion in the direction orthogonal to that of the motion of the Gabor patch as a whole, which makes the patch appear to move in a different direction than it really is moving, influences reaching for the remembered position of the patch with the hand^38^ but not saccades to the patch^37^. In their studies the influence on action always appeared to be much smaller than that on perception, but we attribute this to the way they compare the tasks rather than to a true difference in performance^40^. To make a correct comparison, one should consider that predictions are only needed for the last part of the target’s motion, for which on-line control cannot correct. In our quantitative predictions for the movement errors, we therefore anticipate that the errors in interception will correspond with the part of the influence of any perceptual misjudgement due to the illusion during the last 100 ms before the end of the movement.

The present study shows evidence that clearly contradicts earlier conclusions based on DF^3,4^. Following the two-visual-systems hypothesis one would have expected to find a dissociation between perception and action in MS’s responses to the moving target. However, his errors in interception are completely consistent with his errors in perception. These findings support the alternative interpretation provided earlier^53^ by showing that perception and action are based on the same dorsal processing of motion information. It is the visual attribute that determines whether information is processed in the dorsal or ventral pathway, rather than whether such information is used for perception or for the on-line control of action.

## Methods

### Participants and experimental design

**Fig. 3:**
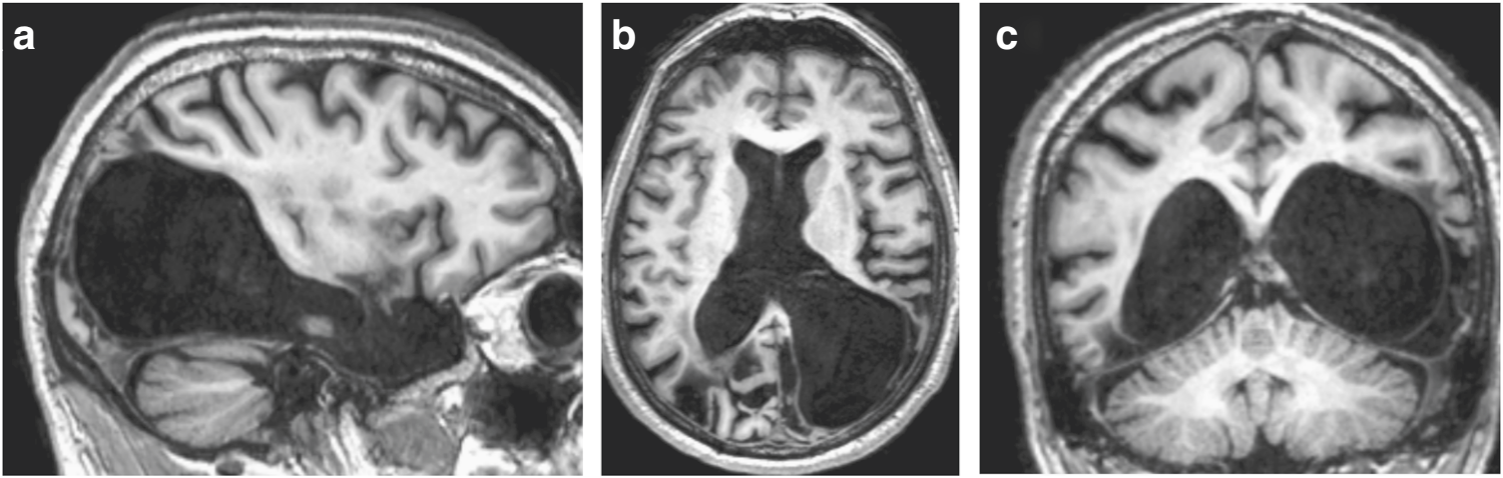
3T anatomical MRI scans of MS’s brain in 2018. Images show the sagittal (**a**), horizontal (**b**), and coronal (**c**) planes of the brain. The left hemisphere has damage to the temporal and occipital lobes, but the parietal and frontal lobes are largely preserved. The right hemisphere damage is much more substantial. The dorsal part of both hemispheres is intact. An extensive and detailed description of MS’s lesion can be found elsewhere^41^

MS is a 65-year-old, left-handed male with bilateral ventromedial damage to his temporo-occipital regions (Fig. 3) due to an idiopathic herpes encephalitis in 1970^41^. As a result, he experiences achromatopsia and left homonymous hemianopia, but his Snellen acuity is normal for both eyes. His other prominent visual disorder is a severe agnosia for objects and faces^42^, but he can read and perceive motion^43^. We compared his performance with that of BK, a 63-year-old, right-handed female with normal vision and without known history of brain damage, as well as with the performance of young participants in a previous study. Those participants, whose data are shown here as additional controls, were one author and nine naïve young subjects (age range 25–34). They were all right handed, with normal or corrected-to-normal vision and without evident motor abnormalities^40^. All participants gave written informed consent. The study was approved by the ethical committee of the Faculty of Behavioral and Movement Sciences at the Vrije Universiteit Amsterdam. The experiments were carried out in accordance with the approved guidelines.

The experiment consisted of two perceptual tasks in which we measured the perceived velocity and the perceived position of a moving target, and an action task in which participants had to intercept similar targets by tapping on them. As both tasks involved motion in the peripheral visual field, a possible ventral contribution to central motion perception^54^ should not play a role. The methods were the same as in the earlier study in which we measured the young controls^40^, except for a few minor modifications that we made to make it easier for MS to participate. We doubled the diameter and increased the contrast of the fixation dot to facilitate fixation. We also used these adjusted values for BK. For MS we also left-right mirrored the stimuli so that the targets moved in his intact hemifield and were easier to intercept with his dominant left hand in the interception task.

MS and BK stood in front of a large screen (Techplex 150, acrylic rear projection screen, width: 1.25 m, height: 1.00 m; tilted backwards by 30° to make tapping more comfortable) onto which stimuli were back-projected (InFocus DepthQ Stereoscopic Projector; resolution 800 by 600 pixels; screen refresh rate: 120 Hz). They had to fixate a 3 cm diameter white dot, 16 cm to the left of the screen center for MS and to its right for BK. The targets were Gabor patches (*σ* = 2 cm, spatial frequency 0.29 cycles/cm) that moved at a constant velocity (40 or 50 cm/s) from right to left for MS and left to right for BK. The gratings within the patches moved at 10 cm/s relative to the Gaussian envelope that defined the patches as a whole. They either moved in the same direction as the patch or in the opposite direction than the patch.

In separate blocks, sequential two-alternative forced-choice discrimination tasks were used to obtain perceptual judgments of position and velocity relative to fixation (Fig. 1a). In the position task, participants had to judge which of two sequentially presented moving patches disappeared closer to the fixation point. Trials started with the fixation point being presented for a random period between 0.5 and 0.7 s, followed by the first of the two moving patches. After a period between 0.5 and 0.7 s, the second moving patch was presented. After these presentations, participants had to report whether the first or the second target ended closer to the fixation point. The researcher recorded the answer by pressing ‘1’ or ‘2’ on a computer keyboard. Once the response was entered, the next trial started. In the velocity task, the sequence of events was the same, but participants had to report which of the two moving patches was faster. In each pair of trials, one patch was the standard, with embedded motion, and the other was a comparison without such motion. The order of the two patches was chosen randomly for each trial.

There were four standard patches. Standard patches were moving with a velocity of either 40 or 50 cm/s, with embedded motion of 10 or −10 cm/s. The presentation time was 700 ms for patches moving at 40 cm/s and 540 ms for patches moving at 50 cm/s. In order to be able to directly compare the effect that the illusion had on perception and on action, we designed the perceptual tasks so that they would reveal participants’ judgments 100 ms before the target reached the fixation position. We chose that moment because 100 ms is the approximate value of the visuomotor delay, the time that it takes to correct an ongoing movement based on new visual information^55,56^. We have reason to believe that how one judges the position and velocity of an object at that moment determines how one will tap on it^57^. Thus, all standard patches disappeared 100 ms before reaching the fixation position. Comparison patches never had embedded motion. In the position task, comparison patches moved at the same velocity as the standard patch but disappeared either at the same position as the standard patch, or 1, 2, or 3 cm further to the left or to the right than the standard patch. In the velocity task, the comparison patches could move at the same velocity as the standard or 10, 20, or 30 cm/s faster or more slowly, always disappearing 100 ms before they would reach the fixation point.

In the interception task we attached an infrared marker to the index finger of the dominant hand and recorded its position with an infrared camera (Optotrak 3020, Northern Digital)^40,57^. Participants started each tapping trial by placing the index finger of their dominant hand on a 3 cm diameter red disk (starting point), 20 cm below the fixation dot. After between 500 and 800 ms a target started moving (from right to left for MS; from left to right for BK). The participants had to tap on the target when it reached the fixation position (Fig. 1b). The targets were the same as the standard patches used in the perceptual tasks, supplemented with targets that had no embedded motion. The targets were visible along the whole trajectory. It took 800 ms and 640 ms for the 40 and 50 cm/s targets to reach the fixation position, respectively. Feedback was provided after each tap. If a target was hit it stopped moving. If the tap was also within the fixation dot, participants got rewarding auditory feedback. If a target was missed, it deflected away from the finger, remaining visible for 500 ms after the tap. MS performed four blocks of 120 trials, with 20 trials for each combination of velocity and embedded motion in each block in random order. BK performed one block of 120 trials. We used more blocks for MS than for BK and the young controls^40^ because MS’s performance was more variable and we observed that many of his trials would have to be rejected, because he frequently either did not move (probably due to his difficulties to follow the fixation instructions given his object agnosia), or made a sliding movement across the screen instead of tapping which made it unclear which position he was aiming for.

### Data analysis

All analyses were performed using R statistical software^58^. To analyse the perceptual tasks we used the ‘quickpsy’ package^59^. Cumulative Gaussian distributions were fit to the proportion of trials in which comparison patches were judged to disappear further than the standard patch, or to move faster. Since MS’s relative position judgments were unreliable (Fig. 2b), and misjudgement of position error previously had a negligible influence on the predicted error^40^, we only used the effect on velocity perception (multiplied by a visuomotor delay of 0.1 s) to predict the effect on tapping. Thus, we made a prediction of MS’s and BK’s errors in interception following the equation (modified from^40^):

$${\mathrm{Error}}_{{\mathrm{predicted}}} = {\mathrm{delay}} \times {\mathrm{error}}_{{\mathrm{velocity}}}$$

We defined the tapping error as the horizontal distance between the tapped position and the position of the center of the patch at the moment of the tap. We removed any biases that were not related to the embedded motion by subtracting the participant’s mean error when tapping on targets without embedded motion (for the same target velocity) from these values. Trials were excluded from the analyses if no tap was detected, if the tapping error was further than three times the standard deviation from the mean for that kind of patch, or if the tap was further than 1.5 cm from the fixation position. This left us with 291 of MS’s 480 trials.

The data of the young controls^40^ were re-analysed using the same equation. In all cases, we calculated 95% confidence intervals for each point of subjective equality (PSE) using parametric bootstrapping with 1000 samples^60^. Once we had the predictions and the actual errors for each standard patch, we averaged across the two velocities (40 and 50 cm/s).

## Data Availability

Data supporting these findings is available^61^ through the Open Science Framework following this link: https://osf.io/kj8fa/.
